# Preclinical evidence of photobiomodulation and clarified açaí in submandibular gland protection during antineoplastic therapy: an experimental study

**DOI:** 10.1007/s10103-026-04876-7

**Published:** 2026-04-30

**Authors:** Wallacy Watson Pereira Melo, Walessa Alana Bragança Aragão, Leonardo de Oliveira Bittencourt, Luciana Eiró-Quirino, Paulo Fernando Santos Mendes, Vinicius Ruan Neves Santos, Hadassa Helez Neves Ferreira, José Messias Perdigão, Hannah Gil de Farias Morais, Herve Rogez, Roseana Almeida Freitas, Manoela Domingues Martins, Rafael Rodrigues Lima, Renata Duarte de Souza-Rodrigues

**Affiliations:** 1 Federal University of Pará, Belém, Brazil; 2 Federal University of Rio Grande do Norte, Natal, Brazil; 3 Federal University of Rio Grande do Sul, Porto Alegre, Brazil

**Keywords:** Chemotherapy, Photobiomodulation, *Euterpe oleracea Mart*—Antioxidant—Submandibular salivary gland, Rat

## Abstract

**Purpose:**

This study aimed to investigate the biochemical and morphological effects of photobiomodulation (PBM) and supplementation with clarified açaí on the submandibular glands in a chemically induced oral mucositis model in rats. Methods: 102 rats were divided into five groups- control: no induction of oral mucositis (OM); untreated group: OM induction, no treatment; PBM: OM induction and laser application (660 nm, 100 mW, 0.24 J, 2.4 s per point, 8 J/cm^2^ and in contact only at the OM site, bilaterally); açaí: OM induction and supplementation with clarified açaí; and PBM + açaí: OM induction and combination of the two treatments mentioned above. Oral mucositis was chemically induced by 5-fluorouracil (5-FU) on days 0 (60 mg/kg) and 2 (40 mg/kg). PMB and supplementation with clarified açaí (dose of 1 ml/100 g body weight) were used from day 0 of the experiment until euthanasia. On days 0 (control group), 8, 10, and 14 (intervention groups), submandibular glands were collected for analysis, with the right side used for oxidative biochemical analysis and the left side for histological analysis. A two-way statistical analysis of variance (ANOVA) was performed, followed by Tukey's post-hoc test (p < 0.05). Results: PMB, alone or in combination with clarified açaí, significantly increased antioxidant capacity levels and showed significantly lower levels of lipid peroxidation and nitric oxide metabolites compared to the control group (p < 0.0001). Morphologically, PBM, alone or combined with clarified açaí, maintained parenchyma, stroma, and acinar structures similar to the control group, which was not subjected to OM induction. Conclusion: It was experimentally demonstrated that PBM, when used alone or combined with clarified açaí, resulted in improved antioxidant response, in addition to providing glandular protection against structural damage caused by chemotherapy.

**Supplementary Information:**

The online version contains supplementary material available at 10.1007/s10103-026-04876-7.

## Introduction

Chemotherapy stands as one of the most widely employed antineoplastic treatments, involving the use of one or more anticancer drugs (chemotherapeutic or alkylating agents) as part of a standardized chemotherapy regimen. It can serve the dual purpose of achieving a curative outcome (often necessitating combinations of drugs) or extending life or alleviating symptoms (palliative chemotherapy). The main goal of chemotherapy regimens is to destroy cancer cells. Its effectiveness is determined by cancer type and stage (Longley et al. 2003). However, certain agents, such as 5-Fluorouracil (5-FU), act intracellularly, releasing inflammatory cytokines that generate oxidative stress capable of causing adverse damage in these regimens, potentially leading to side effects in healthy cells. These damages can significantly impact tissues and organs characterized by a high flow of cell renewal that leads to better functional activity, such as the stomatognathic system (Bachmeier et al. 2019; Thieme et al. 2020). Thus, the salivary glands are one of the most affected structures, and their dysfunction can occur both subjectively (xerostomia) and objectively (hyposalivation) (Bomfin et al. 2017).

Some therapeutic strategies, such as topical mucosal lubricants, saliva substitutes, sugar-free lozenges, pilocarpine, and cevimeline, have been used to prevent or reduce the consequences of salivary gland hypofunction and xerostomia induced by nonsurgical cancer therapies. While these therapies aim to provide greater comfort and alleviate deleterious effects, they offer only limited relief from associated symptoms or may have side effects and contraindications (Villa and Sonis 2020; Mercadante et al. 2021; Golež et al. 2022). In this context, photobiomodulation (PBM) has emerged as a promising alternative. It is a noninvasive, safe, and well-tolerated treatment that involves the use of lasers or LEDs to stimulate biological responses in cells without causing local heating (Arany 2016). PBM can be applied through direct irradiation of the target area or through irradiation of the vascular system. However, the available evidence on PBM for salivary gland dysfunction associated with antineoplastic therapies or xerostomia still lacks studies with well-established protocols (Mercadante et al. 2025).

The use of natural products in prevention and treatment strategies has also been investigated (Yarom et al. 2013; Yarom et al. 2019; Yarom et al. 2020). *Euterpe Oleracea Martius* palm tree, widely known as açaí palm tree, is abundant throughout the soils of the Amazon basin. The fruit is popularly known as açaí and its pulp is abundantly consumed in the form of juice, energy drinks and other products (Alessandra-Perini et al. 2018). The açaí pulp is composed of lipids, proteins, amino acids, total sugars, minor compounds such as fiber and vitamins and various phytochemical compounds, such as phenolics (Alessandra-Perini et al. 2018, Dos Santos et al. 2022, Magalhães et al. 2021). Previous data indicate that açaí, acting on the cells of the oral mucosa of rats, favored tissue regeneration and collagen synthesis at the time of remodeling (Kang and Kim 2018), as well as demonstrating the protective effects of the fruit on intestinal damage, control of oxidative stress, mobilization of defense pathways and tissue repair in an experimental model of chemically induced intestinal mucositis (Magalhães et al. 2021).

Considering both the biostimulatory action of PBM, which can promote local reparative effects, and the systemic antioxidant action promoted by açaí supplementation, the question arose as to whether the combination of both could promote protection or additional improvement of the damage caused by chemotherapy in the submandibular glands. Therefore, the present study aimed to investigate the biochemical and morphological effects of PBM and clarified açaí supplementation, used individually and in combination, on the submandibular salivary glands in an animal model of oral mucositis.

## Materials and methods

### Animal experimental

**Fig. 1:**
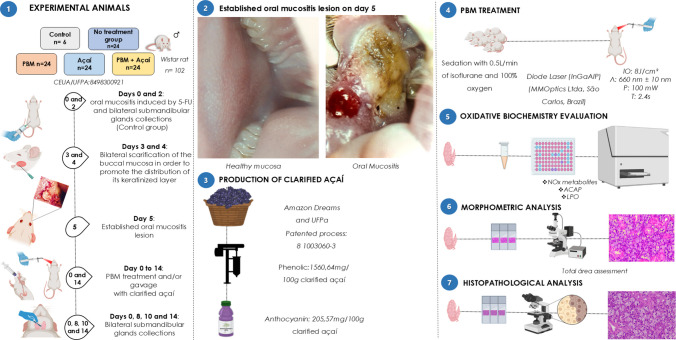
Methodological summary of chemical induction of oral mucositis, collection of glands and analyses. (1) Allocation to experimental groups and flowchart of the experiment; (2) Established oral mucositis lesion on day 5; (3) The stage of production and measurement of the clarified açaí composition; (4) PBM therapy; (5) Oxidative biochemistry evaluation; (6) Morphometric analysis; (7) Histopathological analysis

The experimental procedures were approved by the Ethics Committee for Experimental Animals of the Federal University of Pará, project no. 8498300921, following the recommendations of the NIH Guide for the Care and Use of Laboratory Animals. The steps of this method are summarized in Fig. 1. This study followed the Animal Research: Reporting of In Vivo Experiments (ARRIVE) guidelines (National Research Council).

To perform the sample calculation, a previous study by Thieme et al. 2020 was considered, which uses a methodology similar to that proposed in this article. For this, a loss of 20% was also considered. Thus, the total number of animals was 102, all of which were used in this study and then divided into 5 experimental groups. Thus, Wistar rats (*Rattus norvegicus*), 90 days old, were selected under controlled temperature conditions ranging from 20 to 24ºC, relative air humidity between 40 and 70%, and a 12 h lighting cycle between light and dark. All the rats were fed a standard rodent diet (industrialized feed) and water ad libitum. The animals were obtained from the Central Animal Facility of the Federal University of Pará and acclimatized in the Experimental Vivarium of the Faculty of Pharmacy and Biochemistry at the Institute of Health Sciences. Animals were divided into five experimental groups.

- Control (n = 6): no induction of oral mucositis and animals kept under standard conditions.
- No treatment group (n = 24): induction of oral mucositis, no treatment, and gavage with distilled water.
- PBM (n = 24): induction of oral mucositis and laser application to the buccal mucosa.
- Açaí (n = 24): induction of oral mucositis; food supplementation with açaí clarified by gavage;
- PBM + Açaí (n = 24): induction of oral mucositis and application of PBM associated with dietary supplementation of clarified açaí.

For the submandibular glands collection stage, on day 0, 6 animals from the control group were euthanized. On days 8, 10 and 14, 8 animals from the others groups were randomly euthanized.

### Experimental model of oral mucositis induced by 5-FU

Animals were subjected to the oral mucositis induction protocol proposed by (Sonis et al. 1990), and adapted by Leitão et al. 2007 (Leitão et al. 2007). Oral mucositis was chemically induced by injecting 5-FU (Fluoro-Uracil®500 mg/mL, Sigma-Aldrich, USA) into the peritoneal region at a dose of 60 mg/kg on day 0 and 40 mg/kg on day 2 of the experiment. On days 3 and 4, bilateral scarification of the oral mucosa was performed following the standard protocol for the oral mucositis model in animals by (Sonis et al. 1990), and adapted by Leitão et al. 2007 (Leitão et al. 2007).

On day 5, a blinded histopathologist evaluated all animals to identify the clinical presence of oral mucositis. The main characteristic of the lesion to be observed was the presence of severe hyperemia and erythema, presence of hemorrhage, extensive ulcers, and abscesses, according to what was proposed by Lima et al. 2005.

### PBM treatment

From day 0 to day 14, the animals received daily treatment with PBM. For this, the animals were anesthetized with isoflurane (4–5% Isoflurane, 1 mL/mL, Cristália, Itapira, São Paulo, Brazil) and maintained at approximately 1–2% with vaporized isoflurane in 0.5 L/min of 100% oxygen (Severo et al. 2022). After loss of corneal reflexes, the session was then performed using a diode laser (InGaAIP) (MMOptics Ltda, São Carlos, Brazil) emitting red light with a wavelength of 660 nm (± 10 nm). All parameters used in the PBM were based on the study by Thieme et al. 2020, including the laser application, which was performed intraorally, perpendicular to the oral mucositis lesion, at a central point of the bilateral mucosa. The laser operates continuously with an average power of 100 mW, a spot size of 0.03 cm^2^, and an exposure time of 2.4 s per spot, delivering 0.24 J of energy per spot. The fluence was 8 J/cm^2^, with an irradiance of 3.33 W/cm^2^ and a photon fluence (using eV = 1.9) of 15.2 pJ/cm^2^ or 3.3 Einstein. The device's output power was measured using a power meter. PBM was not applied directly to the submandibular glands, and sessions were always performed in the morning. In the group in which therapies were combined, clarified açaí was administered after PBM to prevent conditions such as regurgitation and irritability during irradiation.

### Production and composition of clarified açaí

*Euterpe Oleracea Martius* fruit juice was prepared according to a patented process (PI 1003060–3, August 4, 2010). Clarified açaí was collected from fresh fruits through a cleaning and pulping process, with the addition of 0.5 L of water per kilogram of fruit. Subsequently, the juice was microfiltered and clarified to obtain a thin, translucent, wine-colored liquid devoid of lipids, proteins, or fibers but rich in phenolic compounds. An aliquot of clarified açaí was characterized according to the composition of total phenolic compounds and anthocyanins, as analyzed by Dos Santos et al. 2022, resulting in a total content of phenolic compounds of 3143.12 mg Eq. gallic acid/L. Through HPLC–DAD methods, the main phenolic compounds of clarified açaí used were: cyanidin-3-glucoside (112.20 mg/L), cyanidin-3-rutinoside (543.30 mg/L), homoorientin (184.15 mg/L), orientin (144.81 mg/L), taxifolin deoxyhexose (13.06 mg/L), vitexin (10.57 mg/L), and isovitexin (10.18 mg/L).

From day 0 to day 14, the animals received intragastric gavage with clarified açaí and distilled water at a dosage of 1 ml/100 g of weight.

### Submandibular gland collection procedures

On day 0 (control group animals only) and on days 8, 10, and 14, the animals in each experimental group were anesthetized with ketamine hydrochloride (90 mg/kg) and xylazine hydrochloride (10 mg/kg) and, after suppression of reflexes, were euthanized by exsanguination. The pair of submandibular glands were collected, with the right side intended for oxidative biochemical analysis and the left for histological analyses.

### Oxidative stress analyses

#### Salivary gland analysis

To analyze the oxidative parameters, the submandibular gland was collected and submerged in a saline solution, subjected to freezing with liquid nitrogen, and stored at −80˚C. For the analysis, the samples of submandibular glands were thawed and resuspended in Tris-20 mM HCl, pH 7.4, at 4˚C, by sonic disaggregation (approximate concentration of 1 g/mL). The supernatant was stored at −80 °C until processing (Dos Santos et al. 2022). Proteins in the samples were quantified to correct the biochemical data using the Bradford et al. method (Bradford 1976). The results were plotted as a percentage of the control.

#### Antioxidant capacity test against peroxyl radicals (ACAP)

This essay measures the response of tissue samples to a reactive oxygen species (ROS)-generating reagent. For this purpose, ROS were produced by thermal decomposition (35◦C) of 2,2′-azobis 2-methylpropionamidine dihydrochloride (ABAP; 4 mM; Sigma-Aldrich, St. Louis, MI, USA). Aliquots of the samples were added to two reading microplates, one with milliQ water and the other with peroxyl radical generator (ABAP) reagent. The method of measuring antioxidant capacity was well explained in Amado et al. 2009, where it is possible to detect the antioxidant capacity of the sample through the concentration of ROS that is equivalent to the fluorescence generated by H2DCF-DA (Invitrogen™, Whaltan, MA, USA) that was used at a final concentration of 40 nM. The higher the fluorescence, the higher the concentration of ROS in the sample. Thus, the samples were analyzed with and without the addition of ABAP to observe the concentration of peroxyl radicals in both situations.

Every 5 min, for 1 h, the samples treated with the ABAP and the samples without this addition (samples with milliQ water) were read in a microplate reader using the fluorescence method (Victor X3; Perkin Elmer, Waltham, MA, USA). To measure the antioxidant capacity, the relative difference in the ROS readings with and without ABAP was considered. Since the results of the method represent the free radicals that are not intercepted, that is, the amount of reactive species that are free and being formed, and this is inversely proportional to the antioxidant capacity, to facilitate the reading and interpretation of the data, we chose to represent the results as the inverse of the relative area, which in fact represents the antioxidant capacity. The antioxidant capacity was calculated according to the equation: 1/(ROSareaABAP – ROSareabackground/ROSareabackground).

The principle of the method indicates that samples with higher antioxidant activity capture a higher amount of peroxyl radicals from ABAP, resulting in lower fluorescence emission. The total fluorescence generated is calculated by integrating the units of fluorescence (UF) over the reading time, after fitting the data to a second-order polynomial function. The results are presented as the difference in the area of ​​UF/min between the samples with and without ABAP, normalized by the area of ​​the sample without ABAP. The inversion of the difference in relative area between the conditions with and without ABAP is used as an indicator of the antioxidant capacity. To avoid false results, the protein concentration was evaluated to standardize the dilution of the samples and subsequent execution of the analysis.

#### Lipid peroxidation assay (LPO)

Malondialdehyde (MDA) levels were measured as an indicator of LPO levels (Esterbauer and Cheeseman 1990). The supernatants and MDA solutions were incubated with a 1:4 solution of methanesulfonic acid: 10.3 mM N-methyl-2-phenylindole diluted in methanol (1:3) at 45 °C for 40 min and read in a spectrophotometer (λ = 570 nm). Results were expressed in nanomoles per microgram (nmol/µg) of protein.

#### Nitric oxide metabolites (Nox)

Nitric oxide metabolites, by products of NO oxidation, were evaluated following the method described by Green et al. 1981. Aliquots of supernatants from submandibular gland were incubated at room temperature for 10 min with equal volumes of Griess solution (1% sulfanilamide in 1% H3PO4:0.1% N-(1-naphthyl)-ethylenediamine dihydrochloride:distilled water, 1:1:1) at room temperature for 10 min. The absorption was recorded at 560 nm and compared with that of standard sodium nitrite solutions. Results were expressed in micromoles per microgram (µmol/µg) of protein.

### Histological analyses

After collecting the submandibular glands, the samples were immersed in 10% formaldehyde for 48 h. Morphometric and histopathological analyses were performed to evaluate morphological and tissue alterations. After fixing the samples in formaldehyde, the glands of each animal were post-fixed in 6% formaldehyde until processing. Afterwards, they were dehydrated in increasing ethanol solutions (70%, 80%, 90%, absolute 1, and absolute 2), cleaned in xylol, and included in Paraplast. Then, twenty sagittal sections of these glands were taken with a thickness of 5 µm and stained with hematoxylin and eosin for morphometry and histopathology, respectively.

#### Histopathological analyses

Two pathologists analyzed the slides qualitatively under blind and independent conditions using a microscope (Eclipse E200, Nikon, Tokyo, Japan; 40 × magnification). Cohen's kappa statistic was applied to assess interobserver agreement, revealing excellent agreement (κ = 0.93). The evaluation followed predefined morphological criteria, including integrity of glandular parenchyma, presence of inflammatory infiltrate, interstitial edema, periductal fibrosis, acinar atrophy, and presence of intracytoplasmic vacuoles. The examiners classified each feature as present or absent, and, when applicable, as mild, moderate or marked, constituting categorical descriptive variables rather than quantitative measurements. Discordant evaluations were reviewed jointly until consensus was achieved. The final classifications were transcribed to a spreadsheet for organizational purposes, and the results were reported as descriptive comparisons between groups at days 8, 10 and 14. Representative photomicrographs were obtained using a color digital camera (Cyber-Shot DSC W-230; Sony, Tokyo, Japan) attached to a microscope (Eclipse E200; Nikon, Tokyo, Japan; 40 × magnification). These images were used solely for illustration and documentation of the morphological findings. The histopathological evaluation itself was performed directly on the slides by the two independent pathologists, and the photomicrographs did not influence or compose the evaluative process.

#### Morphometric analyses

For this analysis, a blinded histopathologist performed the measurement of morphometric tissue evaluation variables, which were expressed in µm^2^ and correspond to the total area of ​​the parenchyma, stroma, and acinus (Hassabou and Elseweidy 2021; Fernandes et al. 2015; Bohl et al. 2008; Merlo et al. 2010). The values ​​of these variables were obtained using a digital image analyzer (ImageJ software, v. 1.53, NIMH of Health, Bethesda, MD, USA; http://rsbweb.nih.gov/ij/). The images were recorded using a color digital camera (Cyber-Shot DSC W-230, Sony, Tokyo, Japan) coupled to a microscope (Eclipse E200, Nikon, Tokyo, Japan; 40 × magnification). Four sagittal sections were randomly obtained from the glands, with an average of four fields per section.

## Statistical analyses

Data were statistically analyzed using GraphPad Prism (version 8.0; San Diego, CA, USA), two-way ANOVA for parametric data, and Tukey's post hoc test, assuming a statistical significance value of p < 0.05. Results are expressed as mean ± standard error (SEM).

## Results

### Oxidative biochemistry evaluation

**Fig. 2:**
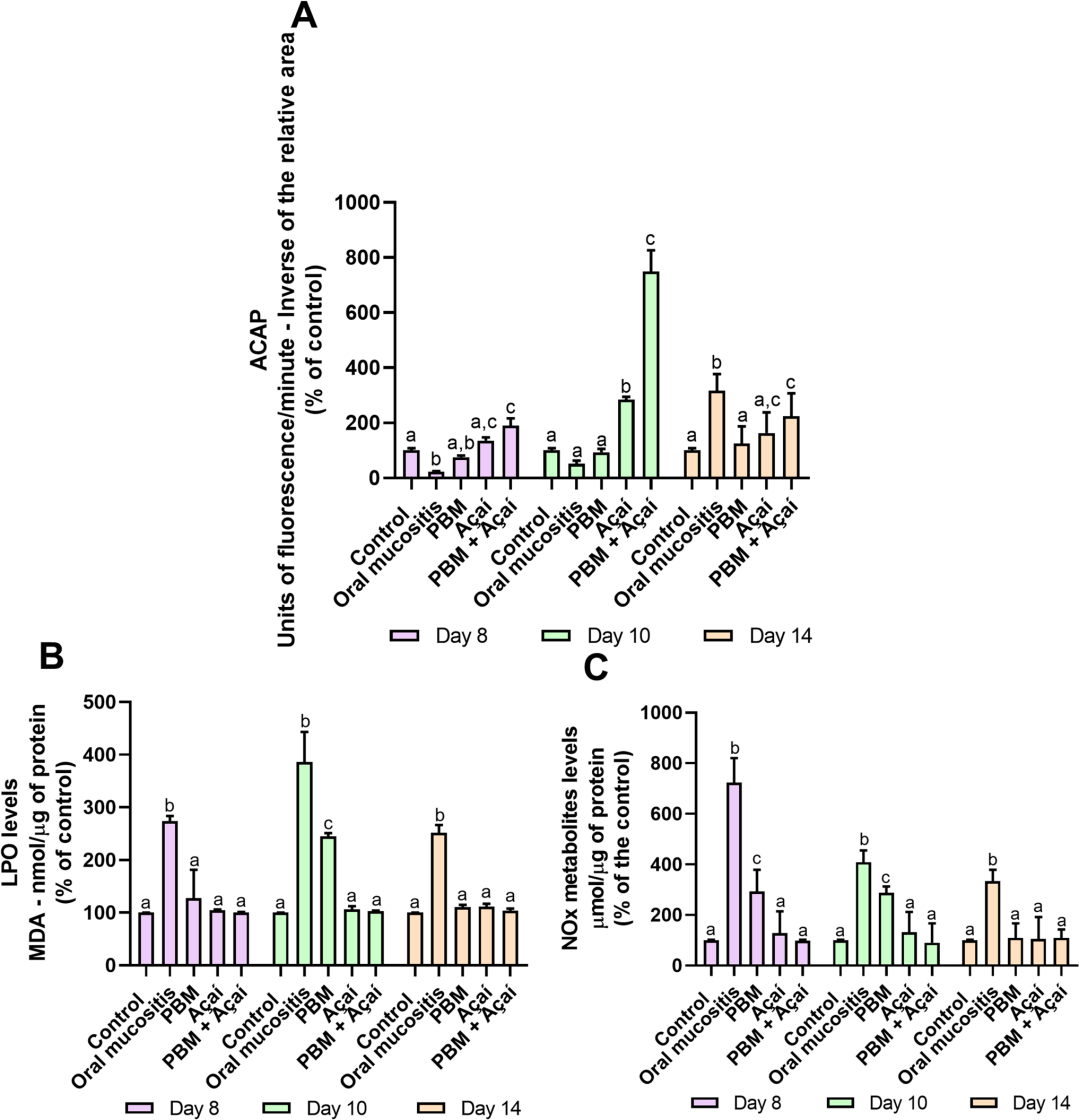
Effects of PBM, Açaí and PBM + Açaí on oxidative biochemistry of the submandibular gland. (A): Antioxidant capacity test against peroxyl radicals (ACAP). The unit of measurement used was unit of florescence per minute (UF/min) between samples with and without ABAP, normalized by the area of ​​the sample without ABAP, thus using the inverse of the relative area and expressed as a % of the control. (B): Lipid Peroxidation Assay (LPO). (C): Nitric oxide metabolites (NOx). The results are expressed as the mean ± standard error of the mean (SEM). Two-way ANOVA for parametric data and Tukey's post hoc test, letters represent statistical differences significance (p < 0.05)

On the 8th day of the experiment, it was observed that açaí administered alone (135.59 ± 11.1; p = 0.0002) or associated with PBM (190.48 ± 25.52; p < 0.0001) significantly increased the levels of antioxidant capacity when compared to the no treatment group (22.18 ± 2.67). On the 10th day of the experiment, the levels of antioxidant capacity remained high and statistically significant in the PBM + açaí group (749.34 ± 76.78), when compared to the no treatment group (51 ± 12.13; p < 0.0001) (Fig. 2A and Supplementary Table 1).

Regarding lipid peroxidation levels, on the 8th day of the experiment, the no treatment group (273.49 ± 10.1) presented significantly higher values ​​than the other groups (PBM: 127.5 ± 54; p < 0.0001; açaí: 104.69 ± 1.3 4; p < 0.0001; PBM + açaí: 100.5 ± 0.9; p < 0.0001). The no treatment group continued to show high levels of lipid peroxidation on days 10 and 14 of the experiment, maintaining a statistically significant difference with the other groups (Day 10—no treatment group: 386.22 ± 56.73; PBM: 244.6 ± 6.38; açaí: 102.89 ± 1; p < 0.0001; açaí: 111.65 ± 5; p < 0.0001; PBM + açaí: 103.56 ± 4; p < 0.0001) (Fig. 2B).

In the case of nitric oxide metabolites, it was observed that, in day 8, the no treatment group (723.52 ± 97) presented high levels (723.52 ± 97) whereas the other groups maintained low levels (PBM: 293.24 ± 85.86; p < 0.0001; açaí: 128.62 ± 86.28; p < 0.0001; PBM + açaí: 99 ± 3; p < 0.0001), with a statistically significant difference. These high levels of nitric oxide metabolites were maintained on days 10 and 14 of the experiment only in the no treatment group, with a statistically significant difference (Day 10—no treatment group: 408.61 ± 46.74; PBM: 287.71 ± 24.8; p < 0.0001; açaí: 132 ± 79.8; p < 0.0001; PBM + açaí: 90.26 ± 76.76; p < 0.0001) (Day 14—no treatment group: 333.78 ± 45.13; PBM: 109.87 ± 57.36; p < 0.0001; açaí: 105.43 ± 86.37; p < 0.0001; PBM + açaí: 109.77 ± 33; p < 0.0001) (Fig. 2C).

### Histological analysis

**Fig. 3:**
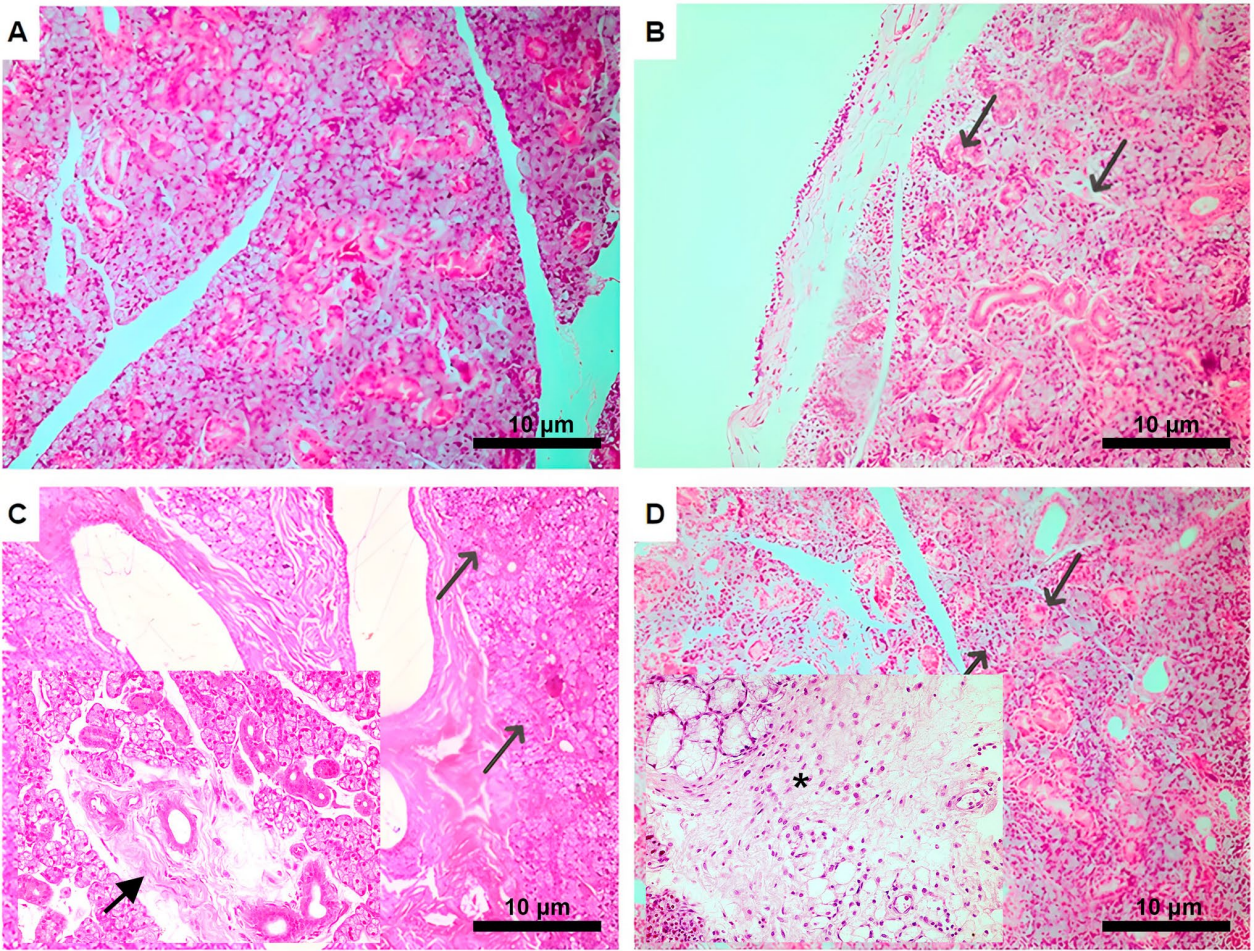
Morphological changes evidenced in the submandibular gland between 8 and 14 days. Representative photomicrographs of (A) control group demonstrating normal glandular parenchyma (H&E, 200x). (B) In the no treatment group, analysis of the main finding in the submandibular gland exposed to chemotherapy at 8 days (H&E, 200x); (⬋) shows a mild mononuclear inflammatory infiltrate in the stroma.(C) Analysis of the submandibular gland in the no treatment group, after 10 days of exposure; (⬈) shows periductal fibrosis (H&E, 200x). In (D), analysis of the submandibular gland after 14 days of exposure in the no treatment group; (⬈) demonstrating intracytoplasmic vacuoles in the stroma and (*) demonstrating scattered mononuclear inflammatory cells (H&E, 200x)

Morphological alterations in the submandibular gland in the experimental groups during days 8, 10, and 14 are shown in Fig. 3. Considering the control group as a baseline for comparison, given the absence of intervention and the presence of normal glandular parenchyma, it was identified that, on the 8th day of the experiment, the not treatment group presented a mild mononuclear inflammatory infiltrate in the stroma, with numerous vacuoles. On day 10, this same group presented periductal fibrosis, acinar atrophy and intracytoplasmic vacuoles. Scattered mononuclear inflammatory cells and intracytoplasmic vacuoles were observed in the stroma on day 14. The other experimental groups revealed similar submandibular salivary morphology to the control.

**Fig. 4:**
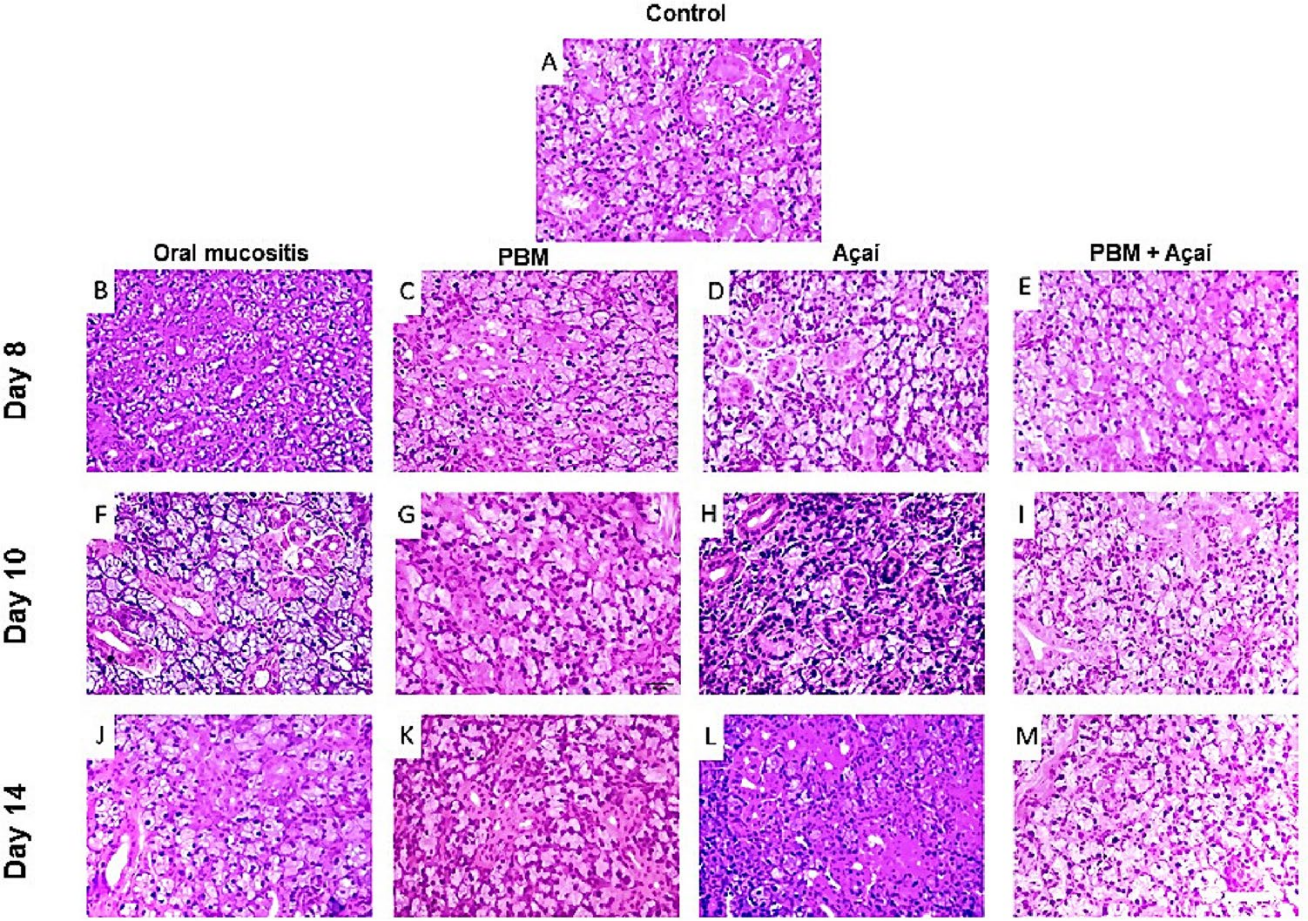
Effect of PBM, Açaí and PBM + Açaí on morphometric analyzes of the submandibular gland at 8, 10 and 14 days. The photomicrographs represent total areas of parenchyma (μm^2^), stroma (μm^2^) and acinus (μm.^2^) (H&E, 400x). (A) Control. Intervention groups in 8 days: (B) No treatment group, (C) PBM, (D) Açaí, (E) PBM + Açaí. Intervention groups in 10 days: (F) No treatment group, (G) PBM, (H) Açaí, (I) PBM + Açaí (H&E, 400x). Intervention groups in 14 days: (J) No treatment group, (K) PBM, (L) Açaí, (M) PBM + Açaí (H&E, 400x)

**Fig. 5:**
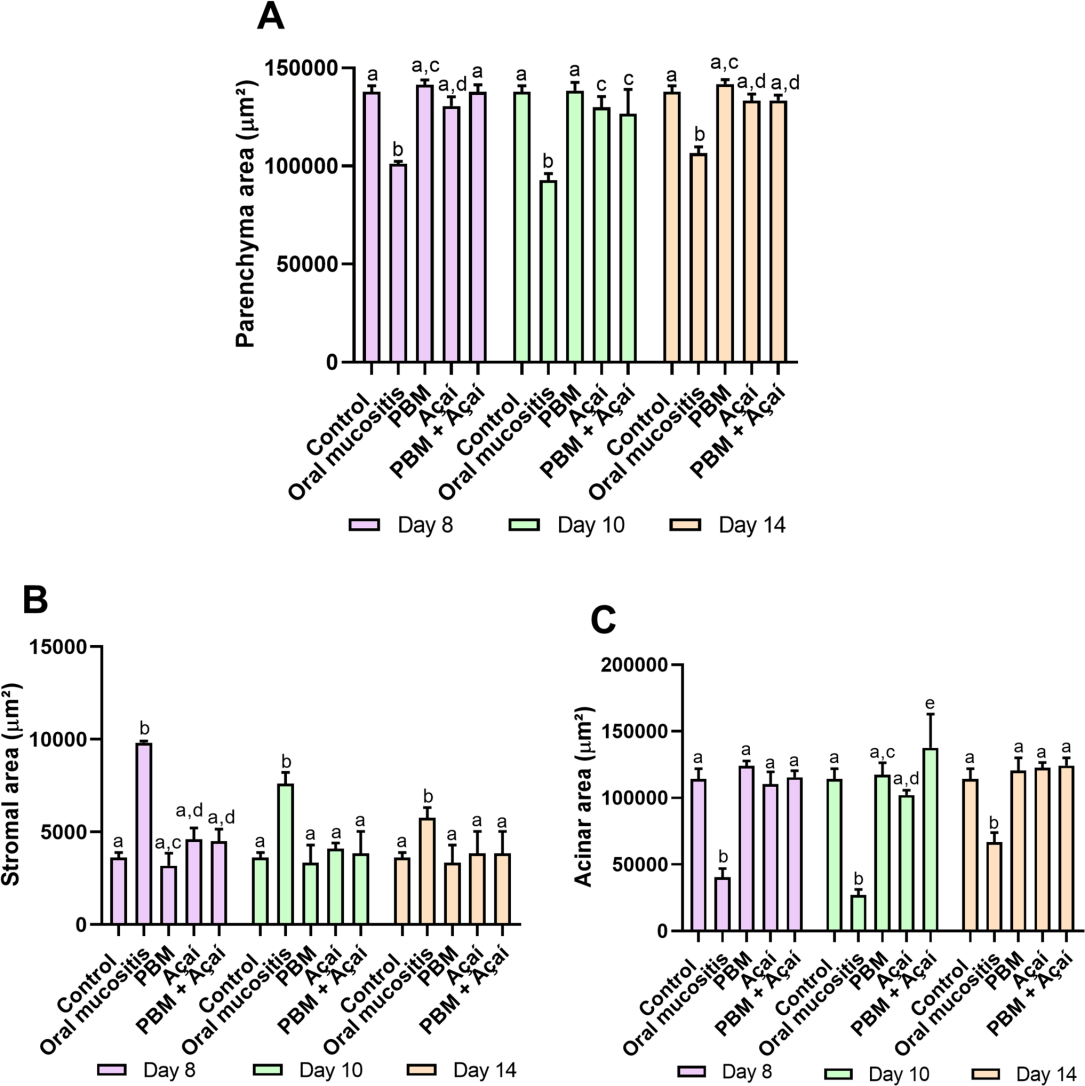
Effects of PBM, Açaí and PBM + Açaí on morphometric structures of the submandibular gland. The figure represents the graphs of the statistical analyzes between the parenchyma, stroma and acinus variables. (A): Total area (μm^2^) in parenchyma (B): Total area (μm^2^) of the stroma (C): Total area (μm.^2^) acini. Anova two way, letters represent statistical differences statistical significance (p < 0.05)

Representative photomicrographs of the morphometric are illustrated in Fig. 4. When evaluating clarified açaí and PBM, it was observed that regardless of whether they were used alone or combined, they maintained the morphological structures of the submandibular glands of the parenchyma, stroma and acinus, presenting values similar to the control throughout the experimental days. In turn, the no treatment group showed a reduction in the structure of the parenchyma, an increase in the stroma and a reduction in acini in the three moments analyzed (Figs. 5A, 5B and 5 C).

## Discussion

The present study aimed to investigate the biochemical and morphological effects of PBM and clarified açaí supplementation, used in combination and individually, on the submandibular salivary glands in a chemically induced oral mucositis model. PBM was performed only in oral mucositis site, and we intended to investigate the possible secondary effect or systemic influence of this treatment considering the anatomical proximity of the mucosa to these glands. Thus, the results revealed that PBM and supplementation with clarified açaí had an important protective effect on the damage caused by chemotherapy in the submandibular glands.

Among the commonly used anticancer drugs, 5-FU was chosen in this study, which is a synthetic analogue of uracil in which it has a fluorine atom in the C-5 position instead of hydrogen (Longley et al. 2003; Bachmeier et al. 2019). This chemotherapy drug has as a secondary effect on the cell the release of inflammatory cytokines, resulting in an imbalance through the increase of pro-oxidant species and reduction of antioxidants, characterized as oxidative stress. Studies show that 5-FU increases the production of ROS, especially the superoxide anion (O2 −), which attack several molecules, mainly the polyunsaturated fatty acids of the cell membranes, resulting in the formation of peroxyl radicals and in a cascade of reactions that culminate in lipid peroxidation and membrane necrosis (Bomfin et al. 2017).

Oral mucositis is characterized by the development of inflammatory ulcers that form in the non-keratinized epithelial mucosa. This condition affects approximately 80% of patients treated with 5-FU, with 20% of these developing severe cases of oral mucositis. Studies demonstrate that salivary gland hypofunction adverse to chemotherapy treatment may be associated with worsening and delayed recovery from oral mucositis (Harada et al. 2022, Hitomi et al. 2019). Furthermore, our choice of the submandibular gland is justified by its predominant contribution to unstimulated saliva and its role in mucosal lubrication and the supply of trophic factors, such as epidermal growth factor, which modulates the pathogenesis and healing of mucositis (Tuner and Hode 2002). Glandular hypofunction associated with chemotherapy can worsen mucositis and delay its recovery (Harada et al. 2022; Hitomi et al. 2019), reinforcing the clinical relevance of preserving the structure of these glands. The pathogenesis and healing of mucositis are strongly modulated by these factors (Melo et al., 2025) 10.1111/jop.70072.

To mediate the effects of 5-FU on these glands, PBM and supplementation with clarified açaí were used. PBM is a promising therapy for protecting glandular structures. The International Society of Oral Oncology (MASCC/ISOO) reported a lack of evidence on recommendations for or against the use of PBM in the salivary glands affected by antineoplastic therapies and highlighted the importance of studies analyzing this therapy to better understand its potential action (Mercadante et al. 2021). An established model of oral mucositis (Sonis et al. 1990) was used to evaluate the possible effects of PBM on the submandibular salivary gland. However, it is important to highlight that the irradiation was not performed directly on the gland, but on the oral mucositis site. In this case, the mechanism of action of PBM appears to be linked to systemic effects possibly resulting from induced molecular changes. We used the term PBM following the nomenclature established by the international consensus (Anders et al. 2015). According to previous studies, the term can refer to local effects resulting from direct irradiation on tissues, and systemic effects resulting from indirect irradiation on body structures, such as blood vessels (da Silva et al. 2023; Dos Santos Malavazzi et al. 2023). In this case, the systemic effects would result from the light (photon)–receptor (acceptor) interaction, which produces molecules that trigger a series of events that interfere in processes such as cell proliferation, viability, differentiation and migration; apoptosis, regulation of signaling and effector molecules, which culminates in the production of effects on tissues, even though indirectly (da Silva et al. 2023). However, the precise mechanism involved in the results obtained in our study could not be identified. We speculate several explanations, but future studies are needed to investigate this issue.

In addition to PBM, supplementation with clarified açaí was also used. Açaí is marketed as a natural dietary supplement and is rich in antioxidant phytochemicals, such as polyphenols (Dos Santos et al. 2022; Magalhães et al. 2021). Among these polyphenols, anthocyanins stand out, which are water-soluble plant pigments of the flavonoid group, responsible for colors ranging from red to violet. According to the findings of this study, exposure to 5-FU increased nitrite and lipid peroxidation levels in the gland. These findings are in agreement with the histopathological analysis, which demonstrated mild mononuclear inflammatory infiltrate in the stroma with many vacuoles. However, the groups supplemented with clarified açaí presented the parenchyma region free of inflammatory infiltrate, similar to the group without exposure. Flavonoids can capture free radicals, which can cause extensive damage to macromolecules such as DNA, and due to their ability to deactivate end products of lipid peroxidation, inflammatory processes resulting from damage to cellular and structural membranes (Alessandra-Perini et al. 2018), as found in our study in the no treatment group. Therefore, flavonoid compounds play a crucial role as systemic antioxidants, with a high capacity to eliminate superoxide anions in addition to inhibiting the production of nitric oxide and its expression. These effects may contribute to the reduction of tissue damage and inflammatory processes, both systemic and local (Alessandra-Perini et al. 2018; Magalhães et al. 2021).

Our findings demonstrated that 5-FU promoted oxidative biochemical damage in these glands with a reduction in 8 and 10 days of antioxidant capacity levels as well as an increase in lipid peroxidation and nitric oxide metabolites levels in all experimental times analyzed. These results are in agreement with those of previous studies (Bachmeier et al. 2019; Bomfin et al. 2017; Ferreira et al. 2022; Murakami-Malaquias-Silva et al. 2023; Fujiwara et al. 2022), which showed that salivary glands are susceptible to changes resulting from 5-FU, which acts directly as a thymidylate synthase inhibitor, and this inhibition results in thymidine monophosphate (dTMP) deficiency (Longley et al. 2003; Bomfin et al. 2017; Ewens et al. 2005), leading to the disruption of DNA and RNA strand biosynthesis and repair during the S phase of the cell cycle. This process causes damage to the genome, cellular apoptosis, and the generation of ROS (Bomfin et al. 2017; Villa and Sonis 2020; Abdelzaher et al. 2022).

The increase in antioxidant capacity observed in the no treatment group on day 14 may reflect a compensatory biological response to the sustained elevation of ROS typically triggered by 5-FU toxicity. Similar adaptive increases in endogenous antioxidant defenses after chemotherapy-induced oxidative stress have been described in other models, where fluctuations in glutathione and related enzymes occur as a delayed protective response (Chiang et al. 2023). Evidence from experimental mucositis models likewise shows that oxidative stress stimulates both inflammatory activity and later attempts at redox compensation (Ceylanlı,et al. 2022; Al-Hoshary and Zalzala 2023).

The results demonstrate that PBM and clarified açaí, when used alone or combined, maintained similar or even higher antioxidant capacity levels when compared to the control group at 8, 10 and 14 days. Meanwhile, lipid peroxidation levels after PBM and clarified açaí were similar to the control group at 8 and 14 days. These findings suggest that 5-FU may induce free radicals capable of promoting structural and permeability changes in cell membranes, causing a reduction in antioxidant levels and glandular damage, followed by cell death. However, we can assume that PBM promoted an improvement in local antioxidant parameters, thus adding to the systemic antioxidant action of açaí (Mazzeo et al. 2012). This action of açaí may be related to the high concentrations of phenolic compounds which, depending on their chemical structures, may possess antioxidant activities. It is important to highlight that in the three time periods analyzed, the no treatment group presented higher levels of lipid peroxidation than the other groups.

Furthermore, we investigated nitric oxide metabolite levels, and the results showed that, at all-time points analyzed, these were elevated in the no treatment group, concurrent with the presence of inflammatory infiltrates observed in the histopathological analysis, while in the clarified açaí and PBM + clarified açaí groups they were similar to those of the control group. A study had shown that inflammatory cytokines stimulate the production of NO, that is involved in cellular cytotoxicity and inflammatory processes (Liu et al. 2005). Specifically, in relation to 5-FU, after its use, the formation of peroxynitrite occurs via the reaction between cells derived from NO and ROS (Szabó et al. 1997) produced by the inflammatory response. This process initiates lipid peroxidation (Rubbo et al. 1994), which increases the migration of inflammatory cells, increasing the inflammatory condition and resulting in tissue damage (Sethy and Kundu 2021).

Regarding morphological changes, a reduction in the total area of the parenchyma was observed, with a consequent increase in the total area of the stroma and a reduction in the total area of the acini. Corroborating these findings, histopathological analysis identified a discrete mononuclear infiltrate in the stroma after 8 days, followed by periductal fibrosis and acinar atrophy after 10 days, and few mononuclear inflammatory cells in the stroma after 14 days. These biochemical observations agree with the morphometric analyses carried out in the no treatment group, revealing a reduction in the levels of the total parenchyma, leading to an increase in stromal components and a reduction in acinar structures. Histopathological analysis also confirmed the presence of discrete mononuclear inflammatory stromal infiltrates, periductal fibrosis and acinar atrophy. The increase in the stroma and the decrease in the parenchyma and acini could be considered as a compensatory attempt to repair damage related to oxidative stress. A study demonstrated that the oxidative stress caused by the chemotherapy process caused a reduction in glandular structures resulting in a reduction in glandular weight and consequent dysfunction of salivary secretion (Takahashi et al. 2014).

This inflammatory response appears to be associated with increased levels of inflammatory cytokines and nitric oxide metabolites in glandular regions, potentially contributing to the decrease in parenchyma and glandular acini. A previous study demonstrated a link between structural changes in acini and reduced mucopolysaccharide levels in the glandular parenchyma (Kawashima et al. 2016, Amano et al. 2012). These results are consistent with another study that demonstrates the action of 5-FU in increasing inflammatory cytokines capable of promoting acinar apoptosis and loss of parenchymal structure, resulting in decreased salivary flow and impairment of the antimicrobial functions of saliva (Pimentel et al. 2022).

As suggested by the results of our study, PBM and clarified açaí, alone or together, could be used as auxiliary therapeutic strategies to protect the submandibular glands from the harmful effects of chemotherapy, and perhaps improve their hypofunction, reducing and alleviating the discomfort caused by this condition. And this would be relevant, since the preservation of the morphological structure of the glands could be one of the factors that guarantees the maintenance of this comfort for individuals for a longer time, something that the strategies currently maintained for a long time, unfortunately.

Our data raises other unanswered questions. We were unable to assess the effects on the secretion and quality of saliva produced by these glands. Therefore, further studies evaluating salivary composition and function may provide important insights into the relationship between antineoplastic therapy, mucositis, salivary glands, and saliva. Furthermore, since our study did not extend beyond 14 days, future studies with long-term follow-up are needed to clarify the temporal dynamics of antioxidant regulation in salivary glands exposed to chemotherapy, as well as to further investigate the actions of systemic PBM and açaí directly on salivary glands exposed to chemotherapy, potentially leading to the formulation of clinical protocols specifically targeting these glands.

## Conclusion

The results demonstrate that the combination of PBM and clarified açaí exerted a protective effect on the submandibular gland of rats undergoing chemotherapy. The combined therapy not only attenuated the biochemical alterations induced by the antineoplastic treatment but also significantly preserved tissue integrity. This beneficial effect was evidenced by an increase in endogenous antioxidant defenses and a reduction in pro-oxidant markers, reflecting the maintenance of the epithelial and connective tissue components of the gland, such as parenchyma, stroma, and acini. These data suggest the translational potential of this combined approach as a preventive and therapeutic strategy to mitigate glandular dysfunction associated with oral mucositis.

## Supplementary Information

Below is the link to the electronic supplementary material.

Supplementary file1 (PDF 205 KB)

## Data Availability

No datasets were generated or analysed during the current study.

## Abbreviations

PBM: Photobiomodulation
OM: Oral mucositis
5-FU: 5-Fluorouracil
ANOVA: Analysis of variance
ARRIVE: Animal Research: Reporting of In Vivo Experiments
ACAP: Antioxidant capacity test against peroxyl radicals
ABAP: Peroxyl radical generator
ROS: Reactive oxygen species
UF: Units of fluorescence
LPO: Lipid Peroxidation Assay
nmol/µg: Nanomoles per microgram
MDA: Malondialdehyde
NOx: Nitric oxide metabolites
µmol/µg: Micromoles per microgram
SEM: Standard error of the mean
MASCC/ISOO: The International Society of Oral Oncology
dTMP: Thymidine monophosphate deficiency
