# Supplementary material for: Preclinical evidence of photobiomodulation and clarified açaí in submandibular gland protection during antineoplastic therapy: an experimental study
Source: Lasers Med Sci. 2026 Apr 30;41(1):84. doi: 10.1007/s10103-026-04876-7 (PMC13132951; doi:10.1007/s10103-026-04876-7)
Supplement: Supplementary file 1 — Supplementary file1 (PDF 205 KB) [file 10103_2026_4876_MOESM1_ESM.pdf]

Preclinical evidence of photobiomodulation and clarified açaí in submandibular gland protection during antineoplastic therapy

Supplementary table I. Parametric results of the biochemical analysis of nitrite metabolite levels (NOx), lipid peroxidation levels (LPO) and antioxidant capacity against peroxyl (ACAP). The results were plotted in percentage of the control. And results of the morphometric analyzes being total parenchymal area, total stromal area and total acinar area (μm^2) of the submandibular gland of rats submitted to a chemically induced OM model. Results were expressed as mean ± standard error of mean. Different lowercase letters in columns (inter-group analysis) denote significant difference ($p<0.05$).

	**ACAP (%)**	**LPO (%)**	**Nox (%)**
**Day 8**			
**Control**	100 ± 7.8[a]	100 ± 0.07[a]	100 ± 2.16[a]
**Oral mucositis**	22.18 ± 2.67[b]	273.49 ± 10.1[a]	723.52 ± 97[b]
**Photobiomodulation**	75.79 ± 5.7[a,b]	127.5 ± 54[a]	293.24 ± 85.86[a]
**Açaí**	135.59 ± 11.1[a,c]	104.69 ± 1.3 4[b]	128.62 ± 86.28[a,c]
**Photobiomodulation + Açaí**	190.48 ± 25.52[c]	100.5 ± 0.9[c]	99 ± 3[a,c]
**Day 10**			
**Control**	100 ± 7.8[a]	100 ± 0.07[a]	100 ± 2.16[a]
**Oral mucositis**	51 ± 12.13[b]	386.22 ± 56.73[b]	408.61 ± 46.74[b]
**Photobiomodulation**	92.98 ± 12.16[a]	244.6 ± 6.38[c]	287.71 ± 24.8[a]
**Açaí**	283.44 ± 11.31[a]	105.93 ± 6.31[a]	132 ± 79.8[a]
**Photobiomodulation + Açaí**	749.34 ± 76.78[a]	102.89 ± 1[a]	90.26 ± 76.76[a]
**Day 14**			
**Control**	100 ± 7.8[a]	100 ± 0.07[a]	100 ± 2.16[a]
**Oral mucositis**	317.18 ± 59.7 1[b]	251.54 ± 14.75[b]	333.78 ± 45.13[b]
**Photobiomodulation**	124.7 ± 61.99[c]	110.21 ± 4.48[c]	109.87 ± 57.36[a]
**Açaí**	163 ± 74.79[a]	111.65 ± 5[a]	105.43 ± 86.37[a]
**Photobiomodulation + Açaí**	223.37 ± 84[a]	103.56 ± 4[a]	109.77 ± 33[a]

Morphometric analyzes

	**Parenchyma área** (μm^2)	**Stromal área** (μm^2)	**Acinar área** (μm^2)
**Day 8**			
**Control**	137,941.42 ± 3,036.63[a]	3,621.67 ± 271.7[a]	114,211.35 ± 7,746.29[a]
**Oral mucositis**	101,159.58 ± 1,338.63[b]	9,814.65 ± 90.7[b]	40,475.28 ± 6,395.82[b]
**Photobiomodulation**	141,455.36 ± 2,512.60[a,c]	3,181.56 ± 679.72[a,c]	124,102.04 ± 3,664.48[a]
**Açaí**	130,517.01 ± 4,839.34[a,d]	4,604.74 ± 619[a,d]	110,257.91 ± 9,357.33[a]
**Photobiomodulation + Açaí**	137,839.94 ± 3,571.53[a]	4,504.74 ± 656.63[a,d]	115,476.84 ± 4,980.73[a]

**Day 10**			
**Control**	137,941.42 ± 3,036.63 [a]	3,621.67 ± 271.7 [a]	114,211.35 ± 7,746.29 [a]
**Oral mucositis**	92,841.21 ± 3,384.34 [b]	7,614.65 ± 598.48 [b]	27,141.94 ± 4,045.12 [b]
**Photobiomodulation**	138,441.27 ± 4,254.20 [a]	3,348.22 ± 944.91 [a]	117,301.44 ± 9,141.72 [a,c]
**Açaí**	129,983.65 ± 5,447.04[c]	4,104.74 ± 296.08 [a]	102,140.54 ± 3,634.82 [a,d]
**Photobiomodulation + Açaí**	126,716.70 ± 124,04.30 [c]	3,838.07 ± 1,196.09 [a]	137,620.15 ± 25,284.56 [e]
**Day 14**			
**Control**	137,941.42 ± 3,036.63 [a]	3,621.67 ± 271.7 [a]	114,211.35 ± 7,746.29 [a]
**Oral mucositis**	106,560.55 ± 3,195.49 [b]	5,771.40 ± 543.80 [b]	66,687.52 ± 7,307.32 [b]
**Photobiomodulation**	141,774.61 ± 2,334.99 [a]	3,348.22 ± 944.91 [a]	120,634.77 ± 9,371.91 [a]
**Açaí**	133,316.98 ± 3,403.04 [c]	3,838.24 ± 1,196.38 [a]	122,544.68 ± 4,102.60 [a]
**Photobiomodulation + Açaí**	133,383.36 ± 2,841.10 [c]	3,838.07 ± 1,196.09 [a]	124,211.35 ± 5,789.19 [a]
